# Pharmacokinetics and Efficacy of a Cyanide-Neutralizing [Mo_2_O_2_(µ-S)_2_]^2+^ Based Metallodrug in NMRI Mice

**DOI:** 10.1007/s00775-026-02153-0

**Published:** 2026-05-25

**Authors:** Linda A. Hancock, Bergthora Eiriksdottir, Sigridur G. Suman

**Affiliations:** 1 Science Institute, University of Iceland, Dunhagi 3, Reykjavik, 107 Iceland; 2 ArcticLAS ehf, Reykjavik, Iceland

**Keywords:** Catalytic metallodrug, Molybdenum-sulfur, HCN-inhalation, Cyanide, Oxythiomolybdate, Thiocyanate

## Abstract

Hydrogen cyanide gas formation and subsequent contribution to inhalation injuries caused by residential and industrial fires is an increasing concern in public health care. Currently used cyanide antidotes are limited in their routes of administration and mechanisms of action. A potential cyanide antidote based on the [Mo_2_O_2_(μ-S)_2_]^2^+ core was assessed for its pharmacokinetic behavior and its efficacy in combating acute cyanide poisoning by inhalation in a mouse model. Pharmacokinetic studies revealed rapid absorption of the molybdenum compound into the bloodstream after intraperitoneal injection, and its complete elimination within eight hours. Prophylactic and therapeutic treatments with the compound by itself, and as a catalytic drug co-administered with thiosulfate, resulted in increased survival at lethal hydrocyanic acid concentrations.

**Graphical Abstract:**

**Figure:**
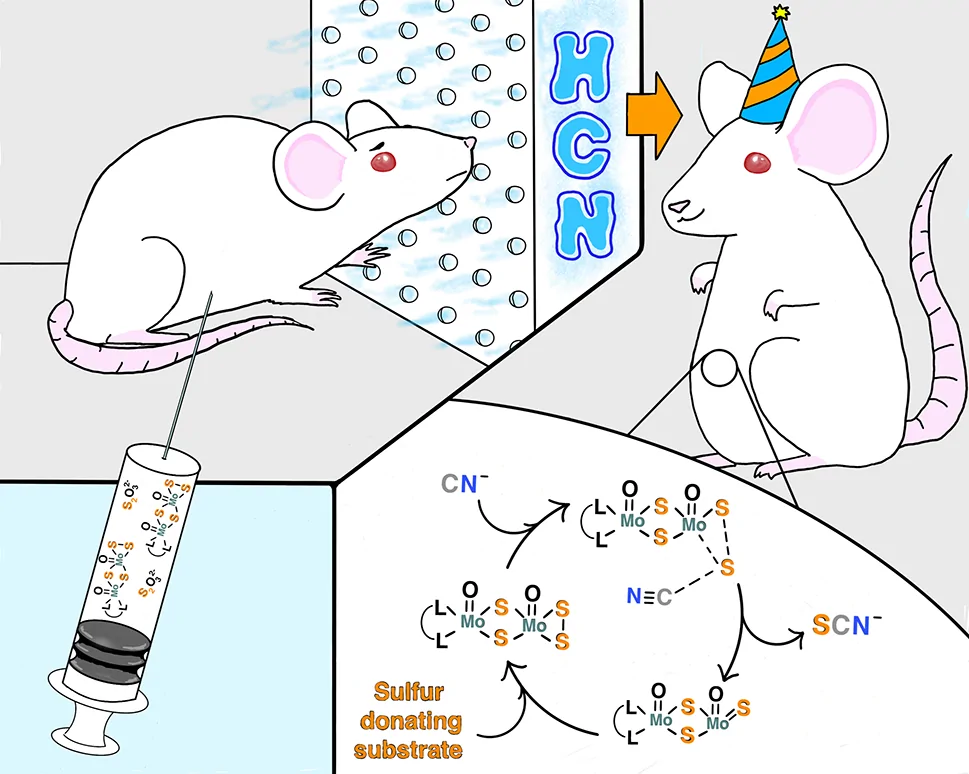

**Supplementary Information:**

The online version contains supplementary material available at https://doi.org/10.1007/s00775-026-02153-0.

## Introduction

Cyanide is used in a wide range of industrial processes including metallurgy and plastics manufacturing; it is also present in smoke from fires [1]. Cyanide occurs naturally in some edible plants such as stone fruits, bamboo shoots, and cassava in the form of cyanogenic glycosides [1, 2]. Its harmful nature to humans and easy access places high importance on availability of effective antidotes towards poisoning by cyanide [3]. Rhodanese is an ubiquitous enzyme whose sole purpose is believed to detoxify cyanide [4]. It has low bioavailability except for in the liver and reacts therefore slowly with ionic water-soluble compounds [4, 5]. Rhodanese functions by using a sulfur-donating substrate—bioavailable sulfur—to convert cyanide to the less toxic thiocyanate [4]. In the event of an accidental cyanide poisoning, whether through natural or anthropogenic sources, where concentrations exceed rhodanese detoxification abilities, an antidote is required. Currently used antidotes approach cyanide intoxication in a few different ways: direct scavenging through chelation of cyanide with metal centers, or indirect scavenging involving substrates affecting natural processes [6–12]. Examples of metal chelation include hydroxocobalamin [13], cobalt-EDTA complex [14], and encapsulated methemoglobin [15]. Indirect scavenging includes nitrite, thiosulfate, and 3-mercaptopyruvate (sulfanegen). The latter two both act as substrates for endogenous cyanide metabolism by rhodanese enzyme [16, 17], and by 3-mercaptopyruvate sulfur transferase (3-MPST) to produce thiocyanate [18] respectively. Nitrite releases NO *in vivo* that was recently shown to antagonize cyanide inhibition, as well as to bind to methemoglobin [19, 20]. Direct scavengers that are efficient have been reported but vary in stability, have high molecular weight, and limited solubility [13, 21–27]. A low molecular weight prodrug, sulfanegen, has been reported to have high efficacy, serving as a sulfur donor to a cyanide sulfurtransferase (3-MPST) that has higher biological availability than the rhodanese enzyme.

**Fig. 1:**
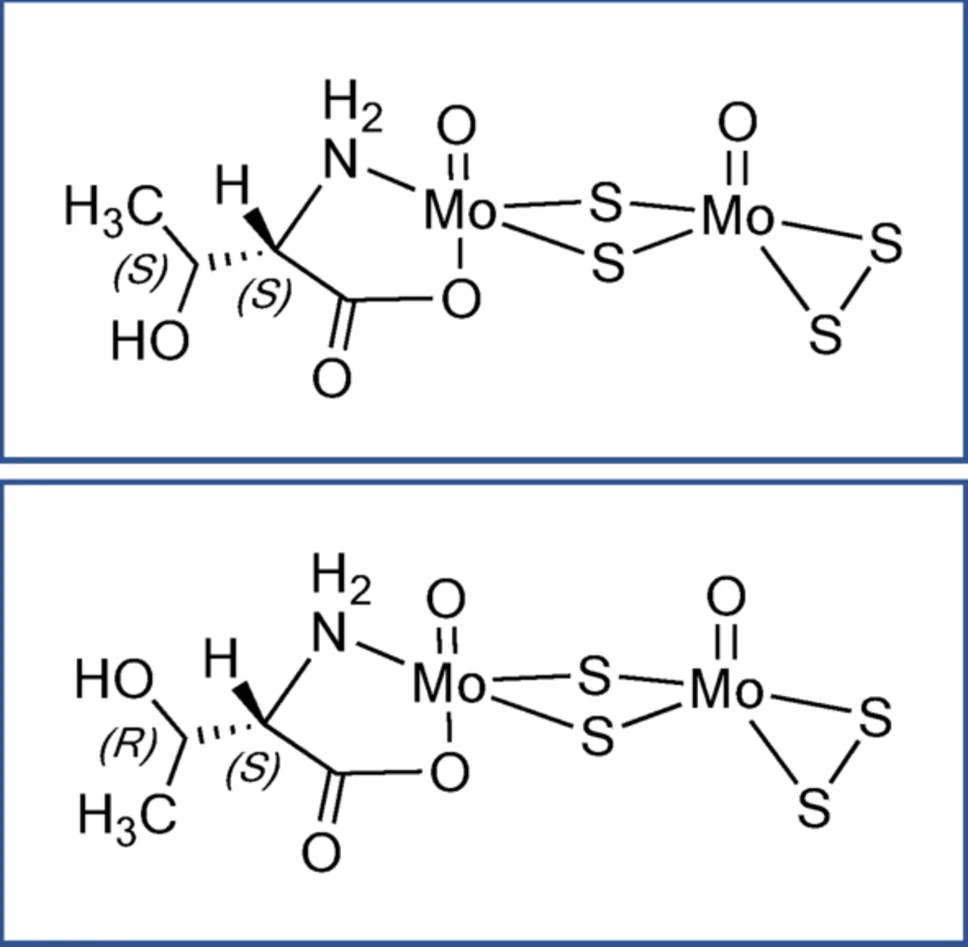
Diastereomers of the *syn*-[Mo_2_O_2_(µ-S)_2_(S_2_)(L-thr)]^-^ anion, **1**

Another route that neither requires participation in metabolic routes nor direct chelation employs molybdenum-sulfur compounds. The Mo-S bonds are well known to react with cyanide to form thiocyanate [28–34], making compounds possessing reactive Mo-S bonds attractive candidates for biological studies and evaluation as potential treatment for cyanide poisoning. Many molybdenum complexes and γ-octamolybdate clusters, with biocompatible amino acid, peptide, or biomimetic ligands, were reported non cytotoxic, as well as binary compounds like MoS_4_^2-^ and a MoS_2_ nanosheet have been reported as non-cytotoxic [35–39].

A class of compounds, including the molybdenum sulfur complex investigated in the present study, exhibit low cytotoxicity, stability in air, water, and acidic solutions [40, 41]. The binuclear *syn*-K[Mo_2_O_2_(µ-S)_2_(S_2_)(thr)] compound, **1**, detoxifies cyanide by directly sulfurating cyanide through its terminal molybdenum-disulfide bond, converting it to the less toxic thiocyanate at physiological pH [3, 41]. It is under study as a catalytic treatment that converts all available cyanide to thiocyanate in the presence of thiosulfate acting as a functional mimic for the rhodanese enzyme (Fig. 1) [41, 42]. Compound **1** is highly water soluble, and compared to rhodanese or 3-MPST it has high bioavailability as it is able to enter cytosol and likely also the mitochondria of the cell [41]. To study clearance of **1** from the bloodstream of this potential cyanide antidote, its pharmacokinetic behavior was assessed over time. Efficacy was evaluated in a series of hydrogen cyanide inhalation exposure studies in a mouse model. Lethal HCN exposure concentrations were verified to agree with reported values in mice [43]. Respiration of the subjects was monitored during the study time periods and recorded. Compound **1** was applied both in a therapeutic and a prophylactic mode of administration by itself, and in combination with thiosulfate as a prophylactic. The results show **1** is able to reduce lethal cyanide toxicity to sub lethal concentrations.

## Materials & Methods

### Reagents

Chemicals were purchased from Sigma-Aldrich and used as received, unless otherwise indicated. KCN (1.0 mM) stock solution was prepared in 40 mM NaOH(aq) to maintain ionized form and stability of the cyanide. H_2_SO_4_ (1.0 M) stock solution was prepared for HCN generation in the inhalation study. NaOH(aq) (40 mM) scrubber solution was prepared as needed in 5 L batches.

### Animal Model

Mice were chosen for the study as they are the smallest mammal feasible for the work. All studies used female NMRI mice (Taconic, Denmark) that were 7–8 weeks old during the study, with body weight ranging from 26 to 35 g. The animals were acclimated for one week prior to the study and received a maintenance diet (Altromin, 1324 from Brogarden), and water *ad libitum*. Veterinary care was provided by ArcticLAS, Reykjavik, Iceland. All experimental protocols complied with the Icelandic Food and Veterinary Authority (MAST), license ref. MAST1504365, MAST1706315, and MAST1712879 and were approved by the National Expert Advisory Board on Animal Welfare. The animal license for the inhalation studies was granted under the condition that the experiments were conducted with anaesthetized animals and that the surviving animals were euthanized at the end of each study.

### Inhalation Chamber

PMMA inhalation chamber was constructed (Merki ehf, Iceland) as 4.2 L, and equipped with an IR heater connected to a thermocouple and an external controller to maintain experimental temperature at 30 °C, a fan for continuous air movement, and three Swagelok ports (Fig. S1). One port was used for injecting KCN solution into a H_2_SO_4_ solution in a 10 ml beaker to generate HCN(g), second port was used for air flow and to inject anesthesia and the third port for HCN(g) sampling and purging of the chamber to the scrubber. The chamber design allows for careful control of *in-situ* HCN generation and concentration and purging and neutralizing of any HCN post experiment. Another inhalation chamber for cyanide gas exposure has been described elsewhere [25].

### Test compound

K[Mo_2_O_2_(µ-S)_2_(S_2_)(thr)].H_2_O; **1**, was synthesized according to published procedure [41]. Four batches of the compound were synthesized and used for these studies. Lyophilization was employed as final purification. Compound purity was verified using ESI-MS: [M − K^+^]^−^, C_4_H_8_Mo_2_NO_5_S_4_ (m/z: 473.7393), found: m/z: 473.7399, colorimetric quantification of thiocyanate yield (as FeSCN^2+^, > 95%) from a reaction of **1** and 10 eq. KCN after 60 min at pH 7.4. Elemental analysis (Midwest Microlab, IN, USA), Calcd. for C_4_H_10_NO_6_S_4_Mo_2_K, C, 9.11, H, 1.91; N, 2.66. Found (representative). C, 8.90; H, 1.78; N, 2.45.

### Pharmacokinetics Study

#### Experimental Design

The uptake and elimination of **1** in the bloodstream was studied by analyzing for molybdenum by MS-OES in whole blood digestates. Whole blood was processed because cyanide is predominantly found in the red blood cells and **1** is easily taken up by cells [44, 45]. For the studies, all treatment solutions of **1** were prepared in sterile phosphate buffered saline (PBS) and filtered with sterile 0.45 μm cellulose acetate (CA) syringe filters. Each study employed four concentration groups: 28, 55, 85, and 170 mg/kg/i.p. The selected time points were identical for each group. Each time point group (*n* = 5) was treated according to its respective dosage group. Treatment solution concentrations were determined by measuring the filtered solutions against the known molar absorptivity coefficient of a stock solution of **1** at 370 nm. Blood was drawn via cardiac puncture under isoflurane anesthesia for whole blood sample collection at their respective time points after which the mice were euthanized under the same anaesthesia.

### Sample Analyses

Whole blood samples were stored in non-heparinized Eppendorf tubes at -80 °C prior to analytical preparation. Whole blood samples were open-air digested in three mL of 70 v/v% ≥99.999% trace metals basis HNO_3_ and one mL of puriss p.a. ≥37 v/v% HCl at > 100 °C until the sample volume would reach approximately 5 mL. Samples were refluxed under a watch glass until a complete digestion was obtained. Digestates were quantitatively transferred into 10.000 ± 0.040 mL volumetric flasks, brought to volume, and filtered through Whatman 0.45 μm CA syringe filters and stored at 4 °C until analysis. Sample preparation quality control included low-volume matrix blanks, method blanks, and laboratory-fortified method blanks. The digestates were diluted 1:5 prior to analysis, leaving a final matrix of 6% HNO_3_ and 2% HCl. Samples were analyzed for molybdenum at 202.095 nm on an AMETEK SPECTRO ARCOS ICP-OES equipped with an ASX-520 autosampler. The instrument parameters are given in Table S1. Quality assurance included blind check standards prepared from a commercially-prepared, certified molybdenum standard (*Trace*CERT, Merck). Calibration checks were performed throughout the analyses for every ten samples as well as at the end of the analyses for quality assurance.

### Statistics

Sample means and standard deviations were calculated for each sample collection time point and dosage group. Suspected statistical outliers were subjected to the Grubbs test at 95% confidence intervals. Samples deemed outliers through the Grubbs test were excluded from the final sample mean calculations. Of the four separate pharmacokinetic experiments, sample means which shared the same dosage group and the same time point were subjected to a one-way analysis of variance (ANOVA) with Scheffé post-hoc tests—all performed at 95% confidence intervals. Paired t-tests were conducted to verify suitability of the data sets for this analysis. F-tests were performed manually in Microsoft Excel and P-values were calculated using GraphPad PRISM software. Sample means determined different from their respective time point groups through failing all the Scheffé post-hoc tests were excluded from the final data compilation. Pooled sample groups were subjected to an additional Grubbs test to obtain pooled sample means. The final data compilation combined all the data from each dosage group and time point to illustrate the mean pharmacokinetic profile for each dosage group. Area under the curve values were calculated using GraphPad PRISM software.

### Inhalation Studies

#### HCN Generation

The inhalation chamber was calibrated for yield of HCN(g) based on the volume of KCN(aq) solution added to a 1 M sulfuric acid solution in a beaker inside the chamber through an injection port. The sampling port was equipped with a Swagelok stopcock and septa that were replaced between samplings. A calibration curve of the “volume HCN generating solution” versus “HCN(g) recovered” was obtained for each standardization (Fig. S2). Two standardizations of the chamber were performed to determine a relative error in sampling; the results of the two standardizations were averaged together for the final chamber calibration curve. The curves were used to calculate the KCN(aq) volumes needed for the desired chamber HCN(g) concentrations.

### Gas Sample Analyses

A series of experiments were conducted to optimize sample collection prior to exposure studies. Various sample collection methods were attempted. The sample collection method which afforded the highest percent recovery and reproducibility was used. To determine actual HCN(g) concentrations, samples were taken during each experiment through a pre-fitted Swagelok sampling port with a 10 mL VICI Series A-2 gas sampling syringe and injected into sealed flasks containing 40 mM NaOH(aq) as the absorption solution to ensure ionization of the HCN to cyanide anion, and to prevent analyte loss by evaporation. Samples were injected into upside-down, sealed 10 mL round-bottom flasks containing 5 mL 40 mM NaOH and which previously had the headspace briefly evacuated via high vacuum. Samples were incubated at 4 °C for 48 h prior to colorimetric analysis for quantification of cyanide as the cyanide anion. All analyses for cyanide were performed spectrophotometrically using a modified version of section D in ASTM D 2036-98 [46]. The method employs the König reaction producing a colored species with absorbance at 481 nm [47]. Colorimetric analyses were performed following the prescribed timing strictly since the colored species is not stable in solution over extended time periods. All cyanide analyses involved a seven-point calibration.

### Statistics

Sample HCN(g) concentrations were determined, and mean concentrations and standard deviations of the same experimental runs were calculated. Survival curves were obtained using Kaplan-Meier [48] plots and LC*t*_*50*_ values were calculated using probit [49] analysis. LC*t*_*50*_ values were also calculated using the sigmoidal curve fitting functionality from the software program GraphPad PRISM 7; using this method, the data point of 0 ppm HCN and 100% survival could be used while probit analysis does not allow for this value. In the first set of experiments, paired t-tests were performed to determine whether a statistically relevant difference existed between beginning sample HCN concentrations and ending sample concentrations. A one-way analysis of variance (ANOVA) was performed on sample mean concentrations belonging to the same experimental group to determine the level of precision and comparability of results. The study groups were treated as experimental variables, hence not subjected to one-way analysis of variance.

### Experimental Conditions

Please see Table 1. Prophylactic and therapeutic experimental scenarios were run. The therapeutic studies were designed to simulate a real-life hydrocyanic acid exposure scenario (such as smoke inhalation) in which the test subjects were exposed to HCN(g) for 10–20 min prior to administration of **1**. Respiration rates were monitored and recorded as this vital sign is a crucial marker for determining the extent of cyanide poisoning [2]. The mice were fully anesthetized with 2.68% v/v isoflurane as determined experimentally. Experimental LC*t*_50_ values were determined and calculated using GraphPad PRISM software. The LC*t*_*50*_ for HCN(g) in the mice was measured under the experimental conditions, as well as the LC*t*_*50*_ for HCN(g) in mice that were pre-treated with 2.6 mmol/kg of thiosulfate.

**Table 1:** Experimental conditions for inhalation studies

Entry	Target [HCN],^a)^ ppm	*N* per group	Exposure time, min.^b)^	Na_2_S_2_O_3_, mmol/kg	Data in Fig.	
*1*	*200*	5	20:20	-	*4a*,* c*,*e*	
*2*	*265*	6	10:20	-	*4b*,* d*,*f*	
*3*	*690*	5	20:20	2.60	*5a*,* c*,*e*	
*4*	*265*	5	20:20	2.60 ^c)^	*5b*,* d*,*f*	
*5*	*650*	6	30^d)^	2.60	*6a-c*	

Target HCN(g) concentration - measured is given in Results and discussion. b) 20:20 and 10:20 are for 20 (10) minute HCN(g) exposure prior to antidote administration of **1**. 30 is for continuous exposure. c) Co-administered with **1** post-exposure. d) Prophylactic co-administration of antidote 15 min prior to HCN(g) exposure

Thiosulfate was administered as a prophylactic in entries 3–5. Its concentration was chosen based on published values as a prophylactic cyanide antidote [50, 51].

*LCt50 Calculations for HCN(g).* Two sets of LC*t*_*50*_ experiments yielded results which are similar to those in the literature. Values calculated for the thirty–minute HCN LC*t*_*50*_ via GraphPad PRISM 7 curve-fitting software in the NMRI mice were 155.2 ± 15.8 ppm (R2 = 0.9552, *n* = 6) and 183.7 ± 5.7 ppm (R2 = 0.9695, *n* = 6) HCN for the first and second experimental set, respectively. Values calculated via probit analysis were 141.43 ± 0.36 ppm (R2 = 0.8426, *n* = 5) and 176.51 ± 0.24 ppm (R2 = 0.9468, *n* = 4) HCN. The Kaplan-Meier survival curve obtained is illustrated in Figure S3.

*LCt50 Calculations with Prophylactic Thiosulfate and HCN(g).* Mice were treated with 2.60 mmol/kg sodium thiosulfate 16–20 min prior to exposure to HCN gas. Both sets of these experiments yielded almost identical results and the Kaplan-Meier survival curve obtained is illustrated in Figure S4. Values calculated for the 30–minute HCN LC*t*_*50*_ via GraphPad PRISM 7 curve fitting software in the NMRI mice were 477.1 ± 0.3 ppm (R2 = 0.9416, *n* = 6) and 455.0 ± 8.8 ppm (R2 = 0.9713, *n* = 7) HCN for the first and second experimental set, respectively. Values calculated via probit analysis were 471.3 ± 0.4 (R2 = 0.8679, *n* = 4) and 445.95 ± 0.22 (R2 = 0.9343, *n* = 5) ppm HCN.

## Results and Discussion

### Test Complex

The test compound **1**, is a chiral compound with threonine possessing two chiral carbon atoms. The synthesis was performed with the *L*-threonine resulting in two diastereoisomers, *R*,* S*- and *S*,* S*- assuming racemization (epimerization) does not take place during the synthesis (Fig. 1). However, it is well established that racemization from *L* to *D* takes place in a first order irreversible reaction at rates dependent on the specific amino acid and external conditions, like temperature, for both free and peptide bound amino acids [52–54]. Although this study did not attempt to separate the isomers, the optical rotation was measured for each batch to have an indication of variance between batches employed in the *in vivo* studies since this may also introduce variability in the results.

**Fig. 2:**
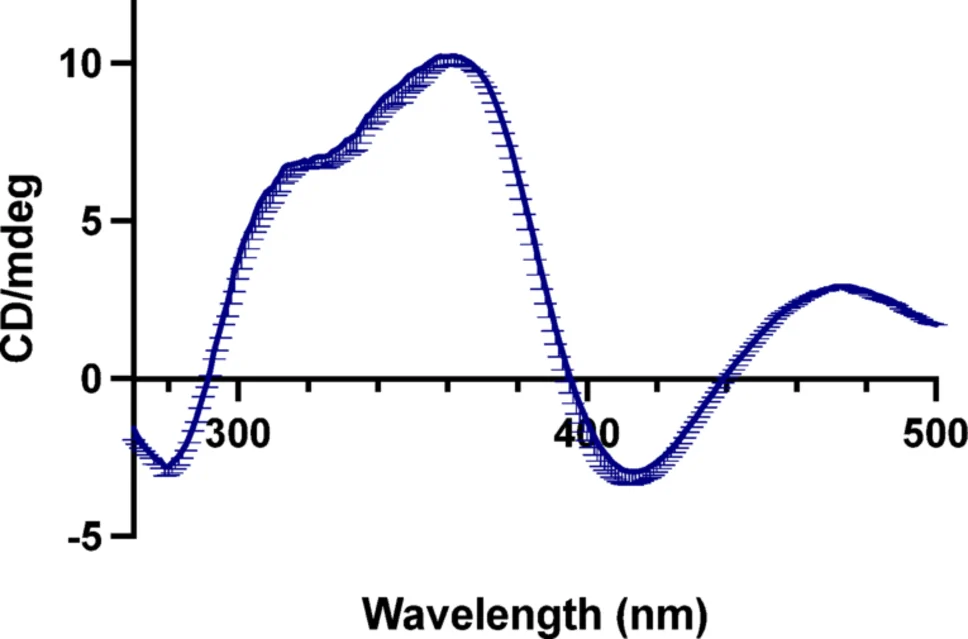
Averaged CD spectrum of batch 1-4 of **1** used in pharmacokinetic studies. Error bars are shown to indicate variance between batches. Measured in DMSO solution

The synthesis of **1** is conducted at room temperature by reacting together a precursor complex and a Fmoc-protected potassium salt of *L*-threonine [41]. No effort was made to optimize an enantiomer selectivity. Figure 2 shows averaged circular dichroism spectra of the batches of **1** employed. Small variations in the graph (shown as error bars) support that structurally the batches are similar. The final purification of **1** was carried out by lyophilization.

The measured optical rotation is given in Table 2. The optical rotation for the four batches averages at 29.2 ± 3.2. This is larger than the error on the measurement (± 0.1) and is likely rooted in the synthesis of **1** resulting in a mixture of diastereomers.

**Table 2:** Optical rotation values for the test complex batches used in the *in vivo* studies

Batch	Optical Rotation,^a^ [a]_D_^24^
*1*	25.9
*2*	29.8
*3*	33.3
*4*	27.6

a) in DMSO Solution

### Pharmacokinetics Data

**Fig. 3:**
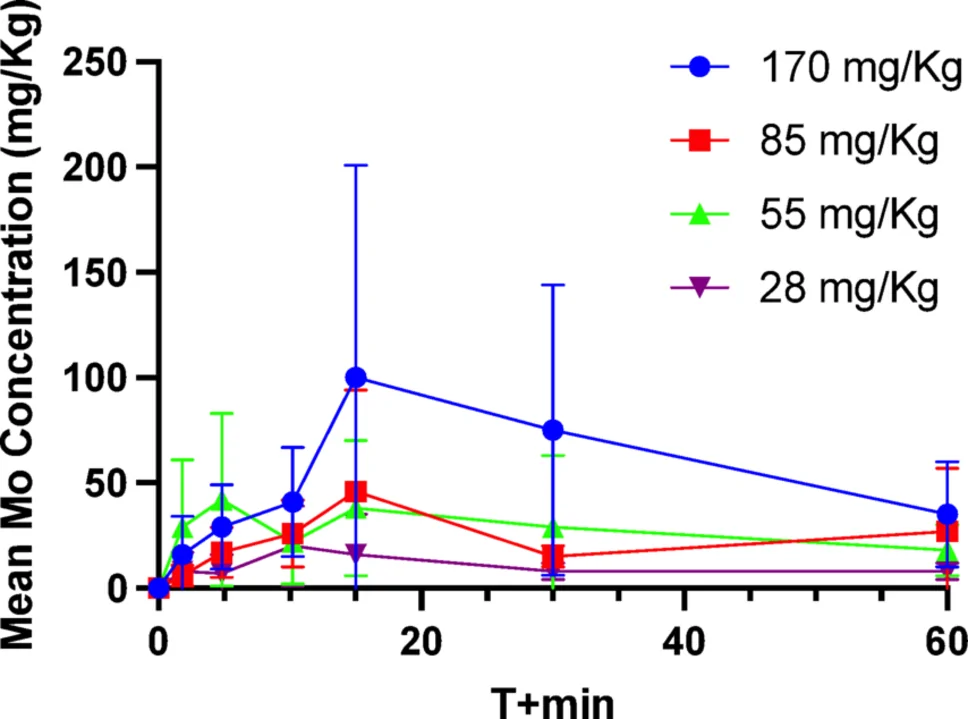
Dose-response curve for the pharmacokinetic studies. See Table 3 for conversion to µmol/kg

NMRI mice are an outbred mouse strain commonly used in many laboratory applications including toxicology and pharmacology. The purposes for using this strain in these experiments include phenotypic variability, avoidance of idiosyncratic, strain-specific resistances/sensitivities, and their docile, relatively easy-to-handle nature. Using an outbred strain does introduce possible high statistical variability in experimental results [55], giving high importance to standardizing environmental, experimental, and analytical procedures. Intraperitoneal injection was chosen as the administration route for the *in vivo* experiments because larger treatment volumes may be used due to its absorption rapidity [56, 57]. Intraperitoneal (i.p.) injection involves injection directly into the peritoneal cavity with an absorption rate which is 25–50% as rapid as intravenous (iv) injection— which provides the most rapid absorption rate [58]. Rapid absorption through intraperitoneal injection is attributed to the robust blood supply in the abdominal cavity [58]. This set of experiments sought out to determine the pharmacokinetics of **1**. This information could be determined by LC-MS/MS [59], however the metabolites of **1** after administration are currently unknown. For this reason, a less cumbersome and more accurate and precise method was chosen for analysis. With the advantage of using a metallodrug as the subject of analysis, it was decided to analyze whole mouse blood for molybdenum via ICP-OES as this would provide a good surrogate profile for **1**.

Molybdenum is not typically present in mammalian blood in amounts detectable by ICP-OES [60, 61], so any detectable molybdenum would be attributed to the presence of **1** and/or its metabolites. When investigating a possible antidote for cyanide poisoning, certain pharmacokinetic features are desirable. Rapid absorption and distribution are important because cyanide is rapidly absorbed and distributed [43]. Without using LC-MS [59, 62] exact metabolites of the drug cannot be determined; therefore, the focus is more on general metabolism, distribution, and absorption as opposed to any possible pharmacodynamic properties. The pharmacokinetic (pk) experiments had four concentration groups that all reached peak bloodstream concentrations between ten and thirty minutes, as shown in Fig. 3; Table 3. This rapid onset of **1** was reflected in the efficacy studies which often gave an improvement in respiration rates at ten to twenty minutes after treatment.

### First Pass Elimination

Consistent occurrence of bimodal distributions in the pharmacokinetic curves for molybdenum may be an indicator of enterohepatic recirculation (Fig. 3) [63, 64]. The reasons for this hypothesis are several fold. Intraperitoneal injection (i.p.), a parenteral route, is known to resemble the pharmacokinetics of oral administration because of its primary route of absorption [57, 65], entering into the mesenteric vessels which empty into the hepatic portal vein directly to the liver [57, 65]. Tetrathiomolybdate (MoS_4_^2−^) has, for example, been found to be rapidly absorbed by the liver in LEC rats, resulting in a biphasic curve for molybdenum serum concentrations [66]. MoS_4_^2−^ has demonstrated a high affinity for hepatocytes [39] which could also explain its hepatic first-pass metabolism *in vivo*. This may be attributed to a general molybdenum metabolism because of high xanthine oxidase distribution in liver tissue across species [67, 68] and it could simply be a regulatory mechanism for molybdenum absorption. Because molybdenum was used as the surrogate marker for **1**, it cannot be determined whether the second peaks observed in the pharmacokinetic curves are hepatic metabolites or excess intact complex entering systemic circulation. It should be noted that if **1** is extensively metabolized in the liver through a similar mechanism as MoS_4_^2−^, monitoring the plasma copper levels could be informative, where an increase in blood or serum copper levels would indicate a mobilization of hepatic copper through complexation [39, 66]. The copper-molybdenum interaction *in vivo* has been well-established [69–71], and molybdates—particularly MoS_4_^2−^ —chelate and mobilize metallothionein-bound copper in hepatocytes as well as complexing copper-bound albumin in plasma [39, 66].

### Bioavailability

To determine the absolute bioavailability for the i.p.-administered complex, pharmacokinetic data for iv bolus administration must be obtained. Bioavailability is described as the ratio of the AUCs of extravascular and intravascular pharmacokinetic curves [72]. This information could be used to determine the extent of the apparent hepatic first-pass metabolism [72] observed in the biphasic pharmacokinetic distributions. By observing the structure of **1** alone, it should have robust bioavailability due to its molecular properties. Although high molecular weight (above 500 g/mol) compounds are often associated with poor bioavailability, there are other molecular properties that have demonstrated a more impactful influence on enteral bioavailability [73]. In a meta-analysis of over 1100 GlaxoSmithKline drug candidates performed by Veber et al., it was found that the number of rotatable bonds and polar surface area had a greater impact on bioavailability in rats [73]. They defined rotatable bonds as any non-cyclic, non-amide, single bonded atom bound to a non-hydrogen atom [73], of which **1** has only three on the threonine ligand. Polar surface areas were calculated using an atom-based method, but it was found that a close correlation existed between these values and the total hydrogen bond count of a drug [73]. Using their parameters for defining the total hydrogen bond count—including hydrogen bond donors and acceptors—it was found that **1** has a total hydrogen bond count of two—both of which are donors. With these molecular properties and the molecular weight of **1**, it should belong somewhere in the fourth quartile of the meta-analysis, with an enteral bioavailability of 39.1%-100% in rats [73]. Intraperitoneal administration of **1** may have a lower bioavailability than the predicted range due to the apparent hepatic first-pass effect, which may be ameliorated through an alternative parenteral route of administration (ROA) such as intramuscular or intravenous injection.

**Table 3:** Pharmacokinetic Study AUC Results

Group	mg/kg (µmol/kg)	T_max_ (min)	C_max_ (mg/kg)	AUC (hrs*mg/kg)
*1*	*28 (54)*	10	20 ± 19	27.2
*2*	*55 (107)*	5	42 ± 41	65.6
*3*	*85 (165)*	15	46 ± 48	69.6
*4*	*170 (330)*	15	100 ± 101	148

### Compound Half-Life

Biological half-life of a drug is described at the time required for a drug’s original concentration (C_*0*_) to reach half of that extrapolated value [74]. This can be calculated simply by using linear regression on a semi-logarithmic plot of the elimination phase of a pharmacokinetic curve (as long as the elimination follows first-order kinetics) [74]. Extrapolated C_*0*_ values, when obtained via intravascular pharmacokinetic data, may be used to estimate the volume of distribution in mice [75]. This would provide further information on the whereabouts of **1** after administration. In general, if a drug is confined to the blood circulation, the volume of distribution value will be around the same as the total volume of blood in the subject; if the distribution volume is the same as the total volume of fluid in the subject, then the drug is thought to be evenly distributed throughout the body; if the volume of distribution exceeds the total water volume of the subject, then the drug is thought to be accumulating in a tissue reservoir, leading to low blood concentrations [75]. This could be particularly useful information considering molybdate half-life values vary greatly across species, dose, and route of administration [69]. Assuming first order elimination kinetics, half-life values were calculated for the 55 mg/kg and 28 mg/kg pooled data groups using linear regression of semi-logarithmic pk plots and the following Equation: _*10*_ ∗ _*p*_ = _*10*_ ∗ _*0*_ − (*k*_el_*t)/2.303 [74]. The elimination phase for both groups was considered to be from the 1 h point to the 6 h point. The half-life for the 55 mg/kg group was 6.8 h with a k_*el*_ of 0.222 h^− 1^. The half-life for the 28 mg/kg group was 5.3 h with a k_*el*_ of 0.229 h^− 1^. Interestingly, the C_*0*_ values for each of the groups were proportional to their initial concentrations: 55 mg/kg C_*0*_ = 21.6 mg/kg Mo and 28 mg/kg C_*0*_ = 9.8 mg/kg Mo.

### Absorption Phase, Distribution Phase, and Elimination Phase

Absorption of the test complex is rapid and variable (Fig. 3). Rapidity of absorption is a desired property for a cyanide antidote, however variability is not. It is for this reason absorption rates and constants could not be obtained. This variability could be due to the test complex’s enantiomeric purity. Bulk microcrystalline solid was used in all of the *in vivo* experiments and although each test batch was well-characterized, the exact chirality of the threonine ligand is currently unknown. Different stereoisomers can greatly impact absorption as well as distribution, metabolism, and elimination [76]. This could also contribute to the bi-phasic absorption and distribution observed in the pharmacokinetic curves. The consistent elimination phases of the pooled 55 mg/kg and 28 mg/kg groups could be due to non-chiral metabolites of **1**, however without adequate elimination phase data from all concentration groups, this is speculative. It could be beneficial to perform a pharmacokinetic study on all four possible stereoisomers of **1** to identify the optimal antidote [76]. Circular dichroism spectra and optical rotation values were obtained for the batches used in the pk experiments. The circular dichroism spectra (Fig. 2) indicate similar structures between the batches. The optical rotation values (Table 2) indicate possible diastereoisomer mixtures with slightly different ratios of the mixture.

### Limitations of I.P. Administration

This pharmacokinetic study was not without its limitations and challenges but provided insight about the biological response of **1** in mice. Although i.p. administration is a parenteral ROA, it provides pk data that closely mimics oral administration [57], and this set of experiments was no exception. Another therapeutic molybdate (MoS_4_^2−^) produced similar biphasic curves after i.p. administration [66], and this could be due to a metabolic response to ingested molybdates for transport to the liver considering the liver’s high xanthine oxidase content in mammals [67]. It is for this reason that future pharmacokinetic experiments involving this class of oxothiomolybdates should involve an alternative parenteral route of administration to avoid the possible first-pass effect.

### Inhalation Studies

Experimental LC*t*_50_ values for HCN were determined prior to conducting the inhalation experiments for suitable target HCN(g) concentrations for the animal model. Prophylactic thiosulfate administration has been reported to significantly increase the LC_*50*_ value for HCN(g) [24, 26]. However, the unusually high values reported were attributed to the specific strain of mice employed. HCN(g) is not commercially available and must be generated *in situ*. Our experimentally determined LC*t*_*50*_ values were found to be 155.2 ± 15.8 ppm for HCN(g) (R^2^ = 0.9552, *n* = 6), and as 477.1 ± 0.3 ppm for HCN(g) with prophylactic sodium thiosulfate (R^2^ = 0.9416, *n* = 6) at 2.6 mmol/kg concentrations. These values were in agreement with previously reported values for mice and rats [50, 77–81]. The survival curves are both very steep, and highly dependent on time. Additionally, treatment breaks were found to increase mortality compared to continuous exposure of same duration. Consequently, a target concentration of 200 ppm HCN was deemed too high for 40 min of total exposure and subsequently a target concentration in the first experimental set was 180 ppm HCN. Further, the recovery HCN(g) concentration was generally around 85% based on KCN(aq). This was commonplace across all inhalation experiments—including the chamber standardization tests. Because the recovered values in the HCN LC*t*_*50*_ experiments fall in line with those of several sources, it is possible that this lower yield is not due to the method used. Several possibilities may affect the gas formation; the reaction does not proceed to completion, HCN(g) is highly water soluble [82, 83] and part of the reaction mixture remains in the sulfuric acid solution, it condenses on the mice’s fur, its distribution in the chamber is not uniform, etc. Regardless of the possible reasons for the poor recoveries, experimentally obtained values for HCN concentrations serve as the sustained values for HCN(g) concentrations in the chamber. Further *t*-statistic tests were performed regarding the sample means between the samples taken in the beginning and samples taken at the end of experiments. These tests revealed a statistical difference between the beginning concentrations and end concentrations of the experimental runs. Assuming a positive-skewed distribution of HCN concentrations over elapsed time, pooled sample means were deemed a good representation of the sustained HCN(g) concentration of the chamber during the mouse experiments.

### Therapeutic Treatment with Compound only

**Fig. 4:**
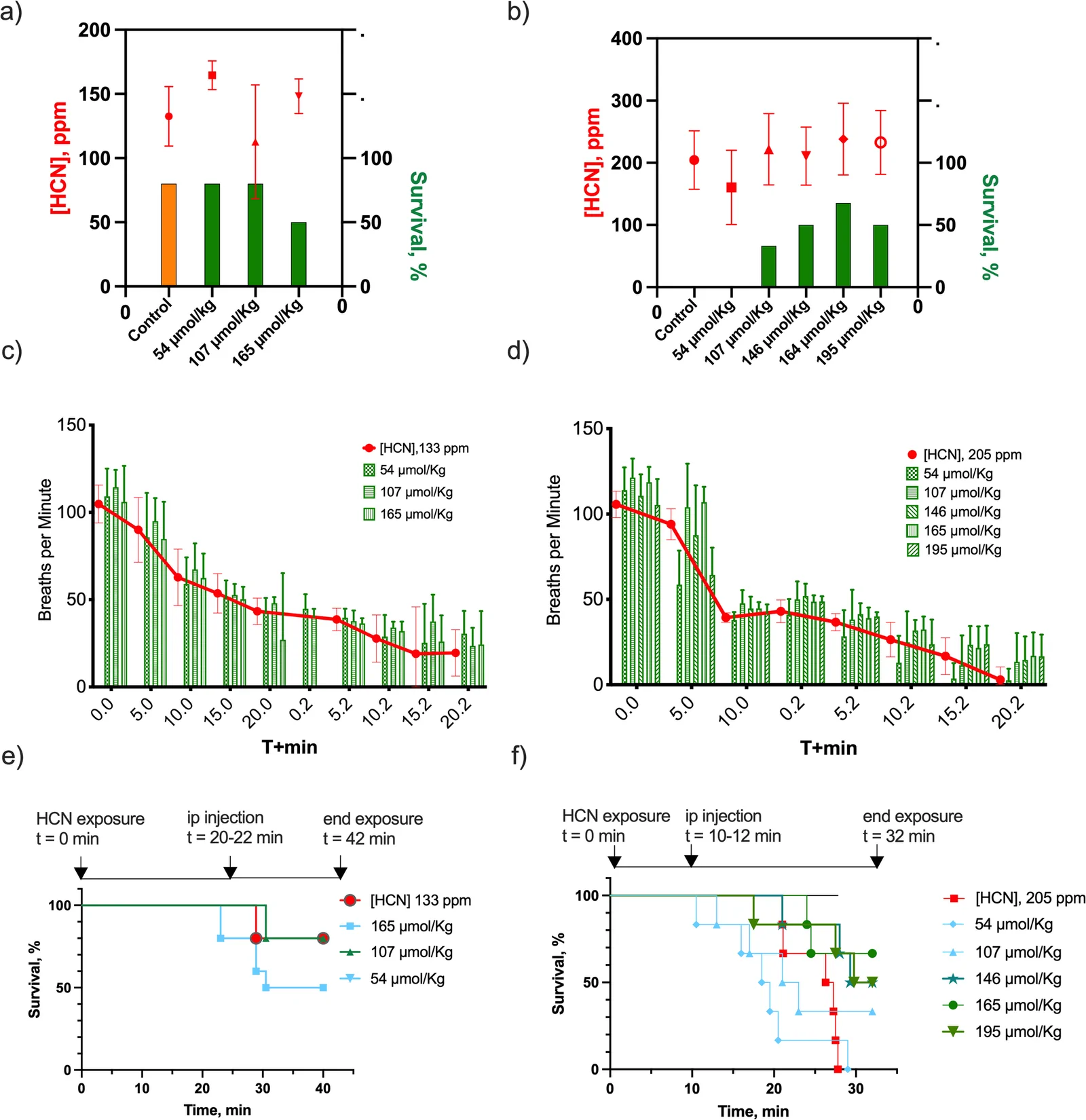
Treatments with **1** only. Sustained HCN concentrations, %Survival, and Mean respiration data for non-lethal HCN(g) exposure (a, c, and e) and lethal HCN(g) exposure (b, d, and f) for control groups and mice therapeutically treated with **1** (Table 1, entries 1 and 2). In (**a**) and (**b**) the columns are labeled with the concentration of **1** employed, and the %Survival shown on the right y-axis in green (*n* = 6). The sustained HCN(g) concentration is shown in red with error bars above each concentration of **1** tested to illustrate the variance in the expected and measured HCN(g) concentration during each experiment. (**c**) and (**d**) show respiration data for the mice as a function of time for the duration of the experiments. Each column represents a specific concentration of **1** and are arranged in order of increasing concentration as shown in a) and b). The red curve is the data for the control group also shown in a) and b). It is given as a curve with error bars rather than a column to clearly display separation between different concentrations of **1**. In (**e**) and (**f**) %Survival is given as a function of time in a common format. It is the same data as given in (a) and (b) plotted to show at what timepoints deaths occurred. Concentrations of **1** that appeared inadequate to improve survival are given in blue, and other concentrations in green, and the control group is given in red. In b) - f) the data do not reflect the observed variance in HCN(g) concentration during the experiments, and therefore (a) and (b) are intended to illustrate that it was not found to be constant

Two sets of experiments were performed with **1** under post exposure conditions (Table 1, entries 1 and 2). The results are given in Fig. 4. The HCN(g) concentration target was set at 200 ppm or sufficiently above the LC*t*_*50*_ value determined to expect sustained HCN(g) concentration above the LC*t*_*50*_ value. Experimental conditions are given in Table 1, entry 1 for Fig. 4 data set shown in charts a), c) and e). Sustained HCN(g) concentration was found near the LC*t*_*50*_ value and consequently non-lethal. The control group was exposed to an average HCN(g) concentration of 132.6 ± 23.2 ppm for forty minutes resulting in 80% survival. It should be noted that a treatment break comparable to the following groups was not implemented for the control group. The average sustained HCN(g) concentration is shown with its error bar along with the %Survival for each group in Fig. 4, graphs a) and b). Each group was found to have different average sustained HCN concentration, as is given in the graphs accordingly. In the non-lethal experiment, 80% survival (one fatality per group) was obtained for all concentration groups except for one where one death occurred immediately after administration of treatment compound (Fig. 4e)). In this case, it was removed from respiration and survival data, but is reported for transparency; effectively two out of four mice survived.

Respiration was monitored in the mice during the inhalation experiments. Anesthesia depth was tested with different concentrations of isoflurane. We found that all mice were fully anaesthetized using 2.68% v/v isoflurane in the chamber. Respiration rates of anesthetized mice at the beginning of the experiment, prior to HCN exposure, ranged from 72 to 126 breaths per minute (bpm). Generally, if respiration rates during anesthesia fall below 50 bpm, the anesthesia is considered too deep, with increased risk of respiratory arrest [84]. Respiration data is shown in Fig. 4c) where the average respiration for the control group (HCN(g), 133 ppm) is given as a curve. There is a slight trend of better respiration in the treated mice versus the control group respiration rates. Despite possible antidotal activity the experimental HCN(g) concentrations used were not optimal. In e) %Survival is shown for all three treatment groups along with the control survival data.

Comparison of the average sustained HCN(g) concentration and survival graph reveals that treatment groups 54 µmol/kg and 165 µmol/kg experienced average HCN concentrations closer to the LC*t*_*50*_ values, and accordingly exhibited earlier fatalities, and lower %Survival in the 165 µmol/kg group. Under these experimental conditions, the mice are mostly able to survive the first twenty minutes of exposure as well as the additional twenty minutes of exposure. This resulted in inconclusive results regarding the efficacy of **1** as a cyanide antidote for this set of experiments. Additionally, paired *t*-tests regarding the target concentrations revealed the mean recovered concentrations were systematically lower than the target concentrations.

In Fig. 4b), d) and e) the experimental parameters (Table 1, entry 2) were changed to lethal conditions; more gas samples were taken to address the precision issues experienced in the first set of experiments, and a control group was employed to determine true lethality of the HCN(g) concentrations used. Two subjects were treated at a time, leading to three separate runs for each set of data with a target HCN concentration of 265 ppm obtained from the second KCN-HCN calibration curve in (Fig S2). The pre-exposure time was shortened to 10 min to avoid loss of subjects before treatment administration. The control group included six subjects exposed to an average concentration of 204.6 ± 47.1 ppm HCN for ten minutes, given HCN-free air during simulated treatment, and re-exposed to HCN for an additional twenty minutes. The control group resulted in zero survival, with all subjects expired within 17 min of the re-exposure time (Fig. 4d) and f)). An ANOVA test of the mean HCN(g) concentrations for each run revealed no statistical difference between the runs (*P* = 0.5629). These experiments revealed clear antidotal activity of **1** in post-exposure to 100% lethal amounts of HCN(g) (Fig. 4b)). The highest survival rate of 67% was with 165 µmol/kg that did not improve by increasing the concentration to 195 µmol/Kg, it in fact decreased to 50%.

Reviewing the respiration data, illustrated in Fig. 4d) there is not a marked difference between the mean respiration values for the mice at any given time interval. However, one of the three mice that had expired in the 195 µmol/kg study did so 15 s before the cutoff time; the other two expired at T + 18 and T + 7 min after treatment while the two mice which expired in the 165 µmol/kg study died at T + 14 and T + 14.5 min.

Considering these factors as well as the limitations of using only six mice for each experiment, these differences in mortality rates might not be significant as the rest of the groups show a clearly logical difference in antidotal activity. As demonstrated in Fig. 4f), the 54 µmol/kg group revealed that this dosage is insufficient for treatment at 100% lethal concentrations of HCN(g); the respiration chart (Fig. 4d)) revealed a markedly lower mean respiration rate than the control derived from the fact that the mice which were treated expired earlier in the experiment than the mice in the control group (Fig. 4f)). This was not explained and is not consistent with the rest of the data.

### Prophylactic Thiosulfate Treatment and Post-Exposure Treatment with **1**

**Fig. 5:**
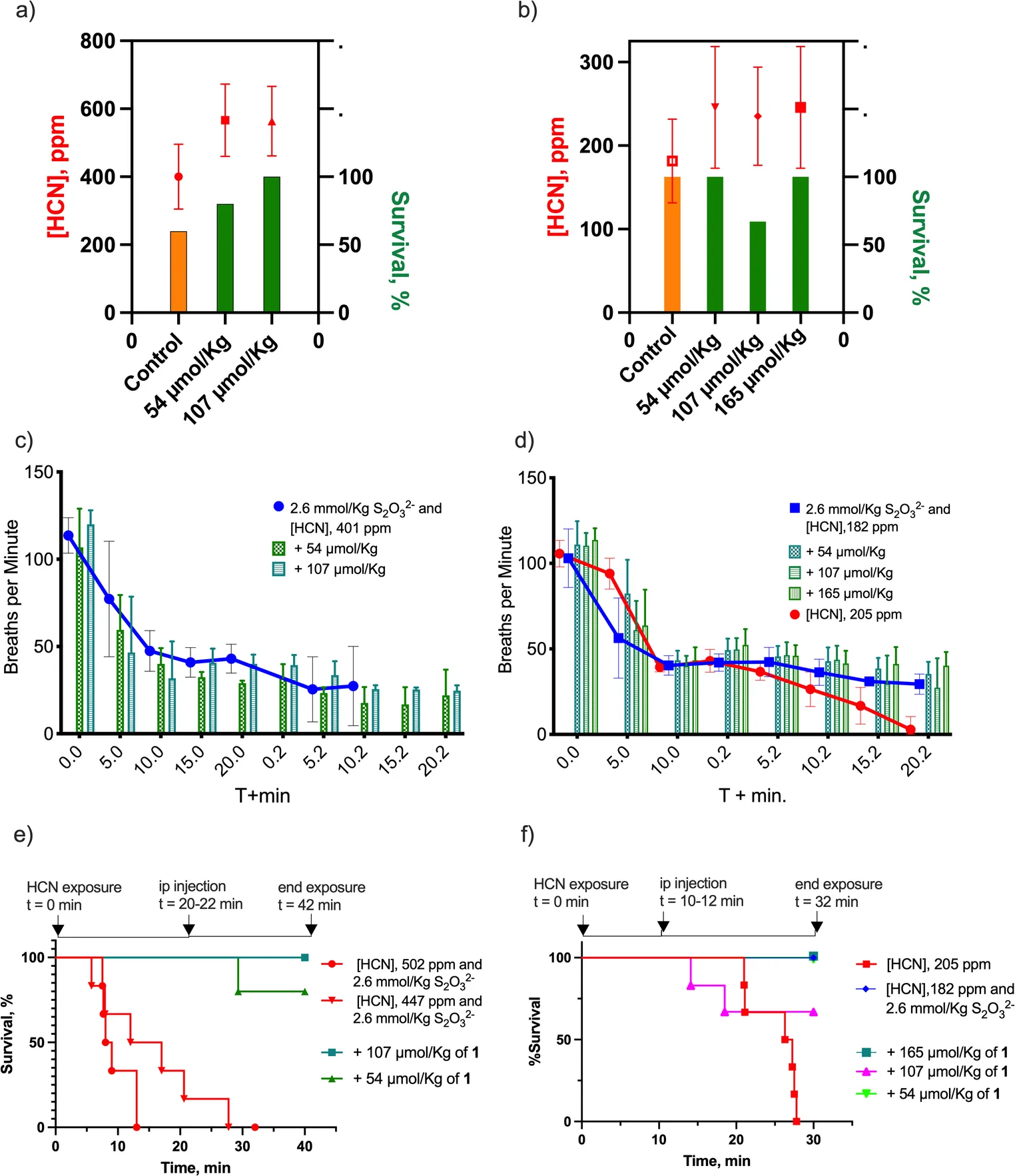
Prophylactic thiosulfate treatment and post-exposure treatment with **1** (a, c, and e) and Combo post-exposure treatment of **1** and thiosulfate (b, d, and f) (Table 1, entries 3 and 4). In (**a**) and (**b**) The sustained [HCN](g) is shown in red with error bars above each concentration of **1** tested to illustrate the variance in the expected and measured HCN(g) concentration during each experiment (left y-axis). The control group in a), c), and e) was treated with thiosulfate 16–20 min prior to HCN(g) exposure. The columns are labeled with the concentration of **1** employed, and the %Survival shown on the right y-axis in green (*n* = 6). Graphs (**c**) and (**d**) show respiration data for the mice as a function of time for the duration of the experiments. Each column represents a specific concentration of **1** arranged in order of increasing concentration as shown in a) and b). The blue curves are control groups (from a), and b)) with thiosulfate treatment where c) is prophylactic, and d) is post-exposure. The red curves in d) and f) are comparative data for an HCN(g) only control groups. They are given with error bars rather than columns to better display separation between different concentrations of **1**. In (**e**) and (**f**) the %Survival is given as a function of time in a common format. It is the same data as given in (a) and (b) plotted to show at what timepoints deaths occurred. Concentrations of **1** that appeared adequate to improve survival are given in green colors, and the control groups are given in red. In (c) - f) the data do not reflect the observed variance in HCN(g) concentration during the experiments, and therefore (a) and (b) are intended to illustrate that it was not found to be constant

Thiosulfate (2.6 mmol/Kg) was administered 16–20 min prior to HCN(g) exposure. The experimental conditions are given in Table 1, entry 3, and in Fig. 5, where the subjects were exposed for 20 min, followed by about 2 min break for compound injection by i.p. administration, and then monitored for 20 min. Positive control experimental results demonstrated the efficacy of prophylactic thiosulfate, extending the LC*t*_*50*_ for HCN(g) under the applied experimental condition to 477.1 ± 0.3 ppm HCN(g) exposure for 30 min (Fig. S4). Results for prophylactic thiosulfate combined with post-exposure compound administration are given in Fig. 5a), c) and e).

The HCN(g) concentrations were measured as before, and the sustained concentration is given with the %Survival for each group. The mean respiration for the group is given along with the mean respiration for the control group that received only prophylactic thiosulfate and, in Fig. 5e), the %Survival is given as a function of time. In Fig. 5a) the sustained HCN(g) concentration for the control group was measured close to the LC*t*_*50*_ of 447 ppm, and lower than the groups with consequent survival of 60%. The two concentration groups were exposed to comparable HCN(g) concentrations of 563.3 ± 102.5 ppm and 566.4 ± 106.4 ppm for 107 µmol/Kg and 54 µmol/Kg respectively. However, two mice in the 107 µmol/Kg group expired before treatment and were not included in the data. 54 µmol/Kg group had one death almost 10 min before the experimental endpoint. The control group included five subjects that were treated with 2.6 mmol/Kg of sodium thiosulfate approximately fifteen minutes prior to exposure to an average concentration of 400.5 ± 95.6 ppm HCN(g) for thirty minutes. Three of the five (60%) mice survived. It should be noted that during this set of control experiments, it was revealed that the chamber had a possible leak. This could explain the poor sample recoveries compared to the treatment experiments which utilized the same volume of HCN generating solution. While the control experiment for this set is not an ideal representative for the actual treatment studies, the last ten minutes of the respiration data for the subjects which were treated with **1** suggest that the respiration was stabilizing and potentially improving for the 54 µmol/Kg, as illustrated in Fig. 5c). It is quite remarkable these four mice survived considering the mean HCN concentrations for these experiments were well over the experimental LC*t*_*100*_ for thirty minutes. The survival data are represented in Fig. 5e).

### Post-Exposure Treatment with Co-administered **1** and Thiosulfate

It is known that **1**’s cyanide conversion rate is bolstered by thiosulfate *ex vivo* [41, 85] and since the prophylactic thiosulfate with post-exposure complex studies from the first set of inhalation experiments revealed inconclusive results, a coadministration with thiosulfate might yield higher antidotal activity of **1** (Table 1, entry 4). Thiosulfate and cyanide react spontaneously when mixed in solution, albeit at a slow rate [17]. This reaction is catalyzed by **1** where the catalytic reaction is initiated with a nucleophilic attack by cyanide on the disulfide ligand of **1** and forming a catalytically active species. This species is capable of neutralizing a lethal concentration of cyanide in less than 20 min at pH of 7.4 [85]. Results from the coadministration experiments are shown in Fig. 5b), d) and f). Figure 5b) illustrates the sustained HCN(g) concentration for four experiments. The control experiment includes treatment with thiosulfate only in a post-exposure scenario (blue line in Fig. 5d). A 100% survival was obtained, explainable by the significantly lower sustained HCN(g) concentration of 180 ppm compared to the parallel runs with 250 ppm, and closer to the LC_***t****50*_ value for HCN(g) of 155 ppm. A second control experiment with 205 ppm HCN(g) concentration only (red line in Fig. 5d) is shown for comparison resulted in zero survival. Graphs 5c) and d) reveal that the mean respiration rates in 5c) are visibly below 50 breaths per minute, and lower than those for the co-administration values in 5d) who maintain an average at 50 breaths per minutes (columns). Lower respiration rates could potentially reduce the total inhaled HCN(g), resulting in higher survival rates. A survival of 100% was surprising considering the sluggish onset of action of thiosulfate [10]. It should be noted the recovered HCN values for the thiosulfate experiments (control) were quite low compared to those of the co-administration studies (Fig. 5b)), resulting in a value close to the LC*t*_*50*_ for thirty minutes. However, it has been established through the course of the post-exposure experiments that the thirty-minute exposure LCs are not the same as those for the experiments in which the subjects received a treatment break. It was found that a much lower concentration is needed for lethality in re-charging the chamber with HCN(g). The volumes of HCN gas generating solution used for the post-exposure experiments were the same as the LC*t*_*50*_ volume in the 30-minute exposure experiments,

yet produced 100% lethality in the control experiment, as shown in Fig. 5f). This further alludes to the behavior of the analyte in the chamber. Throughout the course of the experiments, samples taken at later time intervals were consistently lower than those taken at earlier time intervals. This was deemed not due to loss of the analyte through escape via leaks as this was consistently tested and ruled out. It could be postulated that over time, the HCN is collecting on the mice’s fur or on the floor of the chamber due to its low boiling point. Regardless of the recovery values obtained in the background experiments, the respiration data for post-exposure combination treatment of thiosulfate and **1** (Fig. 5d) show a clear improvement of a crucial vital sign involved in cyanide poisoning compared to the thiosulfate as a prophylactic treatment (Fig. 5c). This would suggest a synergistic effect between thiosulfate and **1** *in vivo*. Thiosulfate clearly assists **1**’s antidotal effects, possibly through serving as a sulfur donor substrate. Comparing left and right panels in Figs. 5 improved respiration and survival is apparent for the post-exposure combination of **1** and thiosulfate.

Comparing left and right panels in Figs. 5 improved respiration and survival is apparent for the post-exposure combination of **1** and thiosulfate.

### Prophylactic **1** and Thiosulfate Treatment

**Fig. 6:**
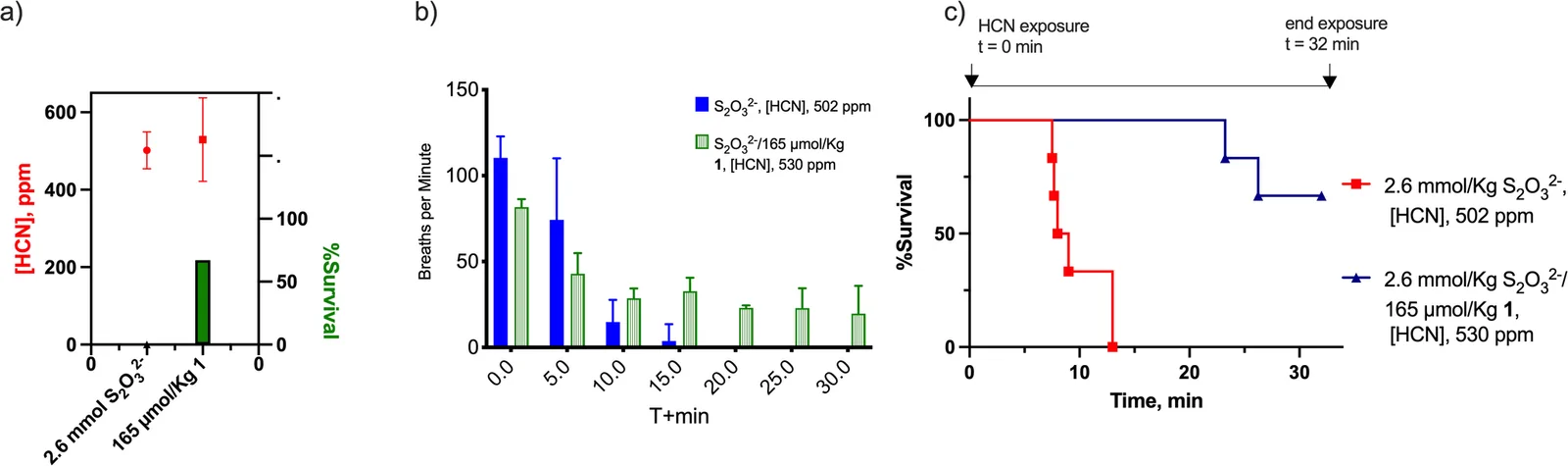
Prophylactic co-administration of **1** and thiosulfate (Table 1, entry 5). (a) Sustained HCN(g) concentration with %Survival for thiosulfate alone, and for thiosulfate + **1**, (b) Mean respiration data as a function of exposure time, and (c) %Survival as a function of time. In (**a**) the green column is labeled with the concentration of **1** added to the given thiosulfate concentration, and the %Survival is given on the right y-axis in green (*n* = 6). The sustained [HCN](g) concentration is shown in red with error bars above each data set to illustrate the variance in the expected and measured HCN(g) concentration during each experiment. (**b**) shows respiration data for the mice as a function of time for the duration of the experiments. A thiosulfate control group and one concentration of **1** + thiosulfate was employed. In (**c**) the %Survival is given as a function of time for continuous HCN(g) exposure for 32 min. It is the same data as given in (a) and (b) plotted to show at what timepoints deaths occurred. The control group is given in red. In b) - (c) the data do not reflect the observed variance in HCN(g) concentration during the experiments, and therefore a) is intended to illustrate that it was not found to be constant

To further investigate the synergistic effects exhibited by the co-administration studies, an exploratory prophylactic study was performed (Table 1, entry 5). The results are given in Fig. 6. The subjects were treated with 165 µmol/kg of **1** and 2.6 mmol/kg sodium thiosulfate fifteen minutes prior to a thirty-minute exposure to 529.7 ± 108.2 ppm HCN. This value is well over the experimentally obtained thirty-minute LC*t*_*100*_ of 502 ppm, which serves as the control group for this experiment. This concentration ensures a 100% lethality concentration if thiosulfate were used alone. Six subjects were used in this study, and four out of the six (67%) mice survived. In the control experiment using the same HCN gas generation solution volume, all six of the subjects expired within thirteen minutes of exposure (Fig. 6c)). With **1**, the subjects that died did so at the 23.15- and 26.15-minute marks. Remarkably, the respiration rates (Fig. 6b)) of the surviving mice were stabilized. It should be noted that the subjects had markedly lower respiration before exposure to HCN(g) compared to thiosulfate alone and their behavioral response to the isoflurane was quite different than to the other experiments. However, this experiment still exhibited a clear synergistic effect between **1** and thiosulfate, as observed in Fig. 6a-c). It is not clear whether **1** and thiosulfate are working directly together or are working in tandem. It is possible however, that **1** is exhibiting biomimetic behavior using thiosulfate as a sulfur donor substrate in a similar fashion as rhodanese.

### Comparison with other antidotes/candidates

Limited data is available for inhalation of HCN in mice and antidotal studies in an inhalation model. HCN has significantly different tissue distribution *in vivo* depending on the exposure route [86–89]. Inhalation for example leads to negligible tissue concentration in the liver, while ingestion leads to high liver concentrations. Tissues with high demand for O_2_ including heart and brain tissue are strongly influenced and they show comparable cyanide concentration profiles for all routes. In contrast, lung and liver tissues show administration-dependent differences in cyanide concentrations with kidneys showing the lowest cyanide tissue concentrations regardless of the administration route [88]. The i.p. route (KCN) gives two to three times higher cyanide concentrations in the liver comparatively and higher tissue concentrations for all routes. The inhalation route (HCN) gives low cyanide concentrations for all tissues, respectively [88]. Similarly, the lethal dose of cyanide varies with the exposure route [3]. These variations complicate comparison with other studies where both the cyanide exposure route and compound administration route play a role. Commercially, hydroxocobalamin (cbl, Cyanokit™), thiosulfate, and thiosulfate/nitrite (Nithiodote™) are available as antidotes via an i.v. route.

An inhalation model study using all male inbred mice, in an inhalation model with a comparable size chamber and group size, evaluated cobinamide (cbi) by itself, thiosulfate alone, and thiosulfate/nitrite combo, as antidotes for cyanide poisoning in a lethal model [26]. Same study also ran experiments with a lethal dose of KCN by i.p. injection (170 µmol/kg) and i.v. injection of treatment compounds. The mice are consistently treated with antidote before exposing to HCN(g) or treated in parallel with i.p. injection of KCN. Effective prophylactic antidotal concentration of “cbi sulfite” is 100 µmol/kg compared to 250 µmol/kg of cbl [26]. Lethal KCN concentration is given as 170 µmol/kg and a stoichiometric relationship is reflected between KCN and cbl (1 eq) or cbi (2 eq.). Similar comparative effectiveness was found for these two in the inhalation model. Cbi was found more effective than thiosulfate alone (2.6 mmol/kg) or when combined with nitrite (1.3 mmol/kg). Thiosulfate/nitrite combo extends 100%Survival beyond the LC_*50*_ for the thiosulfate alone. The reported data for thiosulfate (2.6 mmol/kg) has a very high LC_***t****50*_ of 803 ppm HCN(g) that was attributed the mouse strain used [26]. Regardless, Cbi outperforms thiosulfate by at least 25% even at the lowest HCN(g) concentrations run. Cbi shows efficacy when administered with i.m. injection within 3 min after i.p. treatment with 170 µmol/kg KCN [26]. Cbi is reported efficacious when formulated with 4 eq of nitrite in an i.m. injection and post-exposure model of HCN(g) at 587 ppm [24]. In that experiment coadministration of thiosulfate is used and treatment concentrations of “cbi(NO_2_)_4_” sufficient to reduce the cyanide concentration to sub-lethal and yield 80% survival [26]. Our experimental model could not reproduce any survival at this high HCN(g) concentration without prophylactic treatment with thiosulfate and **1** (Fig. 6).

The present study employed i.p. injection of the antidote after exposure for 10 to 20 min in all cases except for the data shown in Fig. 6 that employed prophylactic treatment with **1** (165 µmol/kg) and thiosulfate (2.6 mmol/kg) and lethal HCN(g). Inhalation LC_***t****50*_ for HCN(g) has been reported as 167 mg/m^3^, that amounts to 151 ppm [89]. In our experiments, prophylactic thiosulfate increased the LC_***t****50*_ for HCN(g) from 155 ppm to 477 ppm, where zero deaths occurred below 400 ppm (Fig. S4). Figure 6 shows that the sustained HCN(g) concentration of 530 ppm resulted in 60% survival after 30 min with **1** and thiosulfate. Thiosulfate alone resulted in 100% deaths in 13 min at sustained HCN(g) concentration of 502 ppm. Direct comparison is therefore moot although it may be said thiosulfate shows similar behavior by increasing HCN(g) concentration tolerance by itself as a prophylactic. As for **1**, it does extend the %Survival to a bit higher HCN(g) concentrations with thiosulfate. It has a dual action as both stoichiometric and a catalytic treatment and further work is in progress. Compared to the Cbi post-exposure data (i.v. injection), **1** also increased survival when the mice were treated with it by itself even after up to 10-minute lethal HCN(g) exposure (Fig. 4b). Comparative data with i.v. injection of **1** and KCN i.p. injection may give a better comparison to commercial or candidate antidotes. However, **1** is uniquely able to conduct the rhodanese enzyme reaction and act as a catalytic treatment.

## Conclusion

The oxothiomolybdate test compound **1** exhibited a clear dose-response. Apart from the 107 µmol/kg groups, the AUC values are reasonably proportional to the concentration given. With intraperitoneal administration there is a risk of hepatic first-pass metabolism [57, 65] and this could be the reason for the bimodal distribution observed in the 107 µmol/kg group. Another contributor to this possible first-pass metabolism could be an affinity for hepatocytes exhibited by **1** since thiomolybdates are known to bind to hepatic metallothionein-bound copper [39, 66]. This metabolic mechanism may also explain the 107 µmol/kg groups’ poor performance in the efficacy studies. The 54 µmol/kg groups have demonstrated it is not a sufficient concentration for the level of HCN exposure involved in this set of experiments. With not much difference in the results between the 146, 165, and 195 µmol/kg treatment groups, the lesser of the three may be the optimal therapeutic concentration for acute cyanide exposure.

Regardless of its possible metabolic challenges, **1** demonstrated efficacy in countering cyanide poisoning. Its performance alone with the concentrations used in these experiments is not superior to 2.60 mmol/kg of sodium thiosulfate. **1** may require an alternate vehicle and route of administration. Its performance is bolstered with the presence of thiosulfate as a sulfur-donating agent where **1** clearly outperforms itself alone showing prophylactic capabilities when co-administered with thiosulfate in the presence of high concentration (530 ppm) of hydrogen cyanide gas. With demonstrated capability of **1** improving respiration rates during cyanide poisoning, it clearly neutralizes the toxicant *in vivo*.

## Electronic Supplementary Material

Below is the link to the electronic supplementary material.

Supplementary Material 1

## Data Availability

Raw data for the pharmacokinetic study and HCN(g) data sets from inhalation experiments may be made available upon request and with permission from MV ehf (grant holder). All other data is included within the paper and the Supplementary Information.

## Abbreviations

ICP-OES: Inductively-coupled plasma-optical emission spectroscopy
3-MPST: 3-mercaptopyruvate sulfurtransferase (EC 2.8.1.2)
PMMA: polymethylmethacrylate
LEC rats: Long-Evans rats with a cinnamon coat color
LC*t*_*50*_: Time dependent lethal median exposure concentration of a toxin

## Declarations

### Notes

HCN is a very toxic gas and precautions must be taken to ensure safe handling of sample processing and disposal.

### Supporting information

ICP-OES instrument parameters. Figure of the inhalation chamber, HCN(g) recovery curves, and Kaplan Meier survival plots for HCN and for HCN/thiosulfate.
