# Supplementary material for: Pharmacokinetics and Efficacy of a Cyanide-Neutralizing [Mo2O2(µ-S)2]2+ Based Metallodrug in NMRI Mice
Source: J Biol Inorg Chem. 2026 May 25;31(6):489–505. doi: 10.1007/s00775-026-02153-0 (PMC13645865; doi:10.1007/s00775-026-02153-0)

Pharmacokinetics and Efficacy of a Cyanide-Neutralizing $[Mo_2O_2(\mu\text{-}S)_2]^{2+}$ based Metallodrug in NMRI Mice.

Linda A. Hancock[1], Bergþóra Eiríksdóttir[2], and Sigridur G. Suman[1]

[1]Science Institute, University of Iceland, Dunhagi 3, 107 Reykjavik, Iceland, [2]ArcticLAS ehf, Reykjavik, Iceland

Corresponding author: Sigridur G. Suman; sgsuman@hi.is

Supplementary Information

Table of contents

Table S1 - ICP OES instrument parameters
Figure S1 - Inhalation Chamber
Figure S2 - Calibration of theoretical vs measured HCN(g) concentration
Figure S3 - Inhalation Chamber HCN LC_{t50} calibration curves
Figure S4 - Inhalation Chamber HCN/$S_2O_3^{2-}$ LC_{t50} calibration curves

Table S1. ICP-OES Instrument Parameters.

Coolant Flow	13	L/min
Auxiliary Flow	0.8	L/min
Sample Flow	0.8	L/min
Pre-Flush	30	s
Normal Time	45	s
Line Width	0.001	
Region Width	0.15	
Plasma Power	1400	W
Pump Speed	30	rpm
Mo Wavelength	202.095	nm

Figure S1. Inhalation Chamber design.
The lid is not show on for clarity

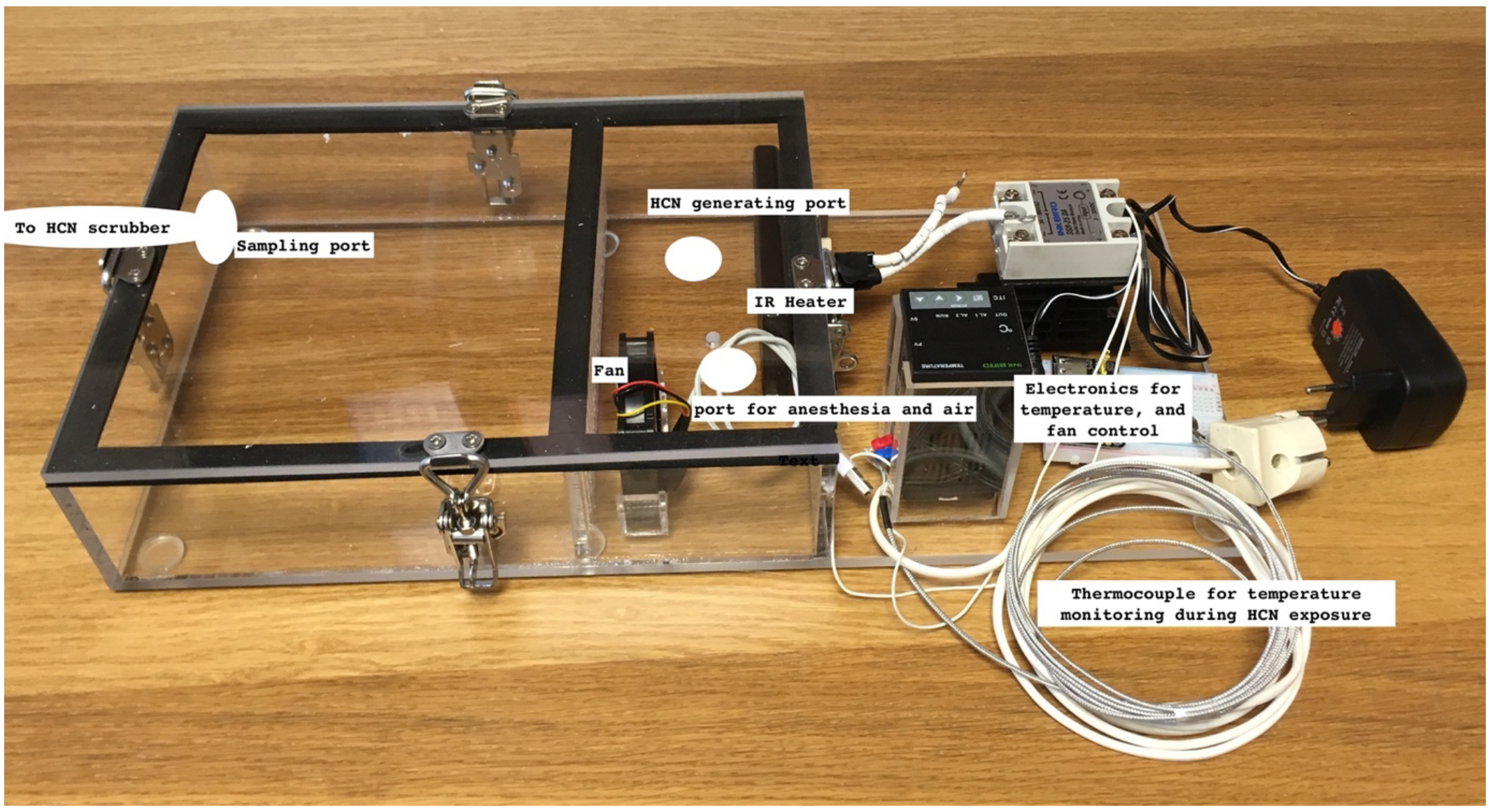

Figure S2. Theoretical vs Measured HCN(g) recovery.

KCN-HCN calibration curves obtained from the chamber standardization experiments

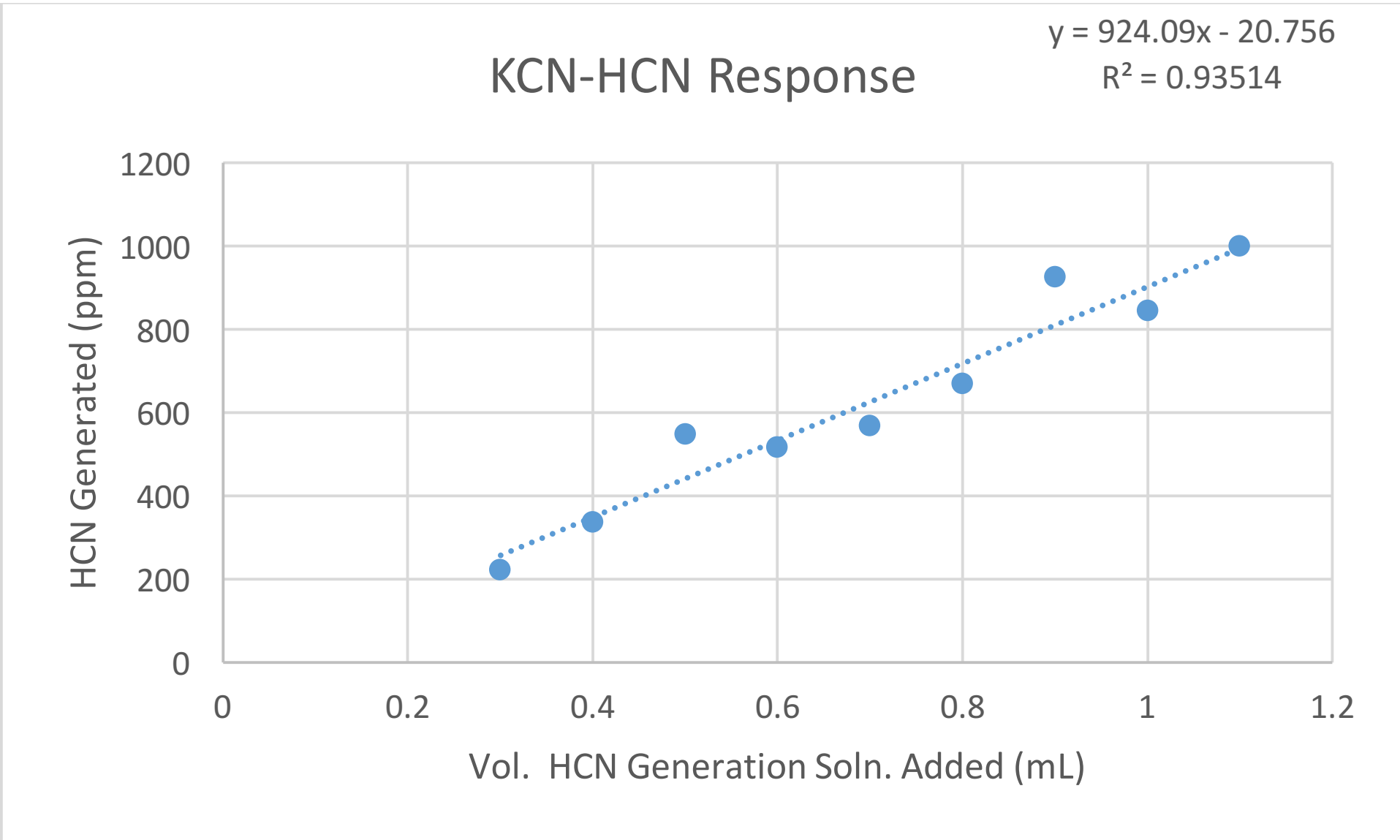

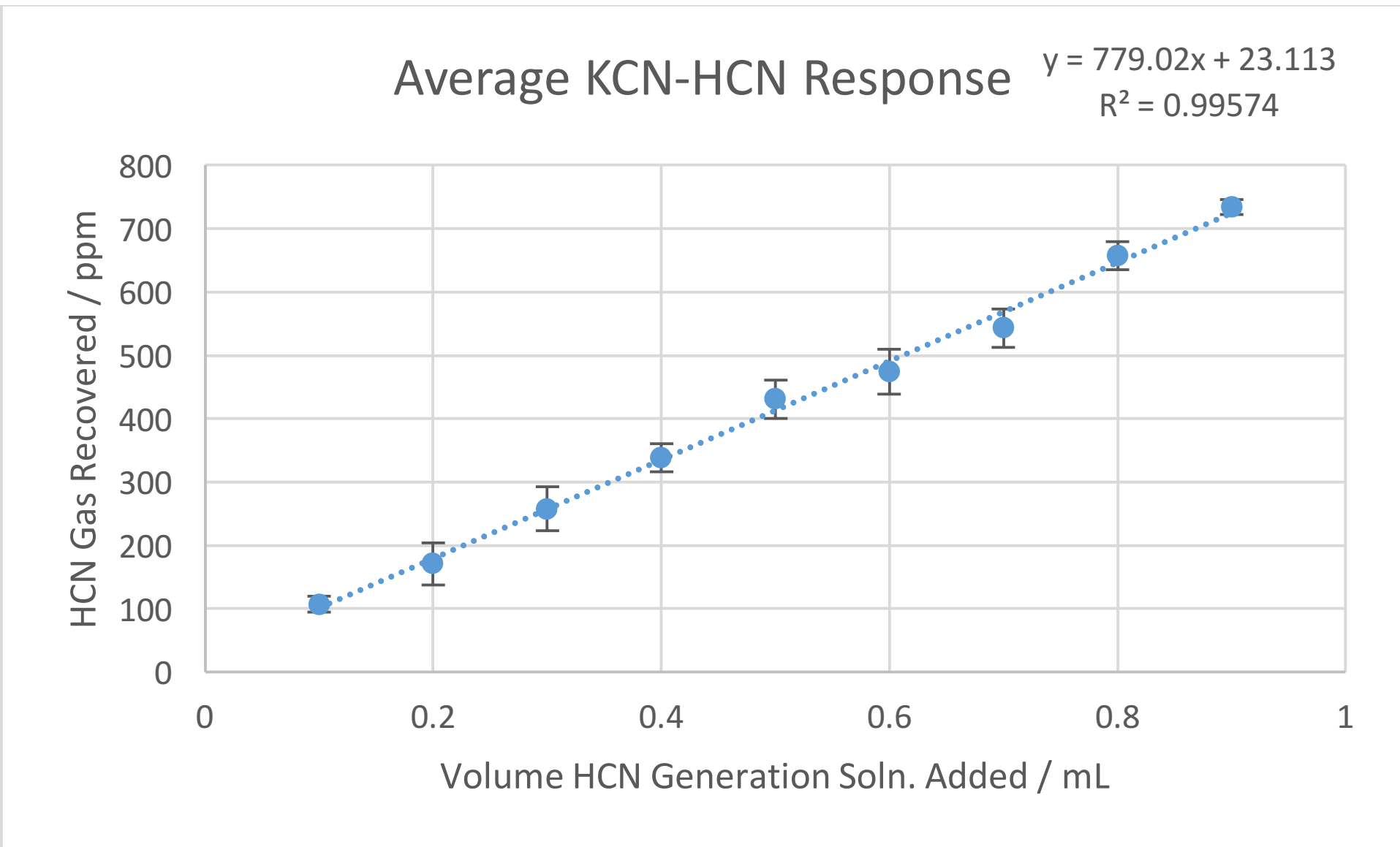

Figure S4. Survival vs Hydrocyanic acid + 2.60 mmol thiosulfate prophylactic inoculation concentration at 30 minute exposure time.

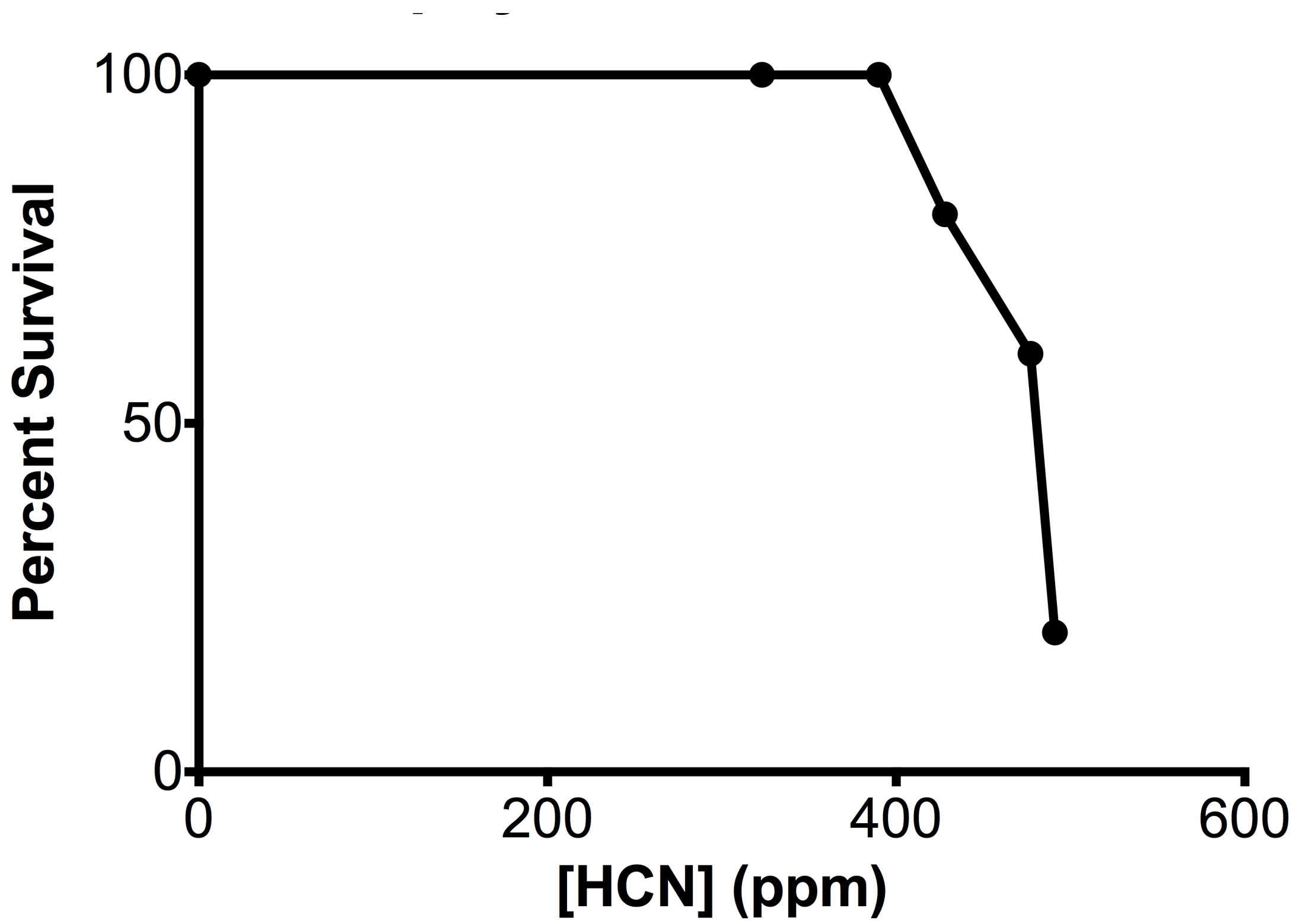

Figure S3. Survival vs Hydrocyanic acid gas concentration at 30 minute exposure time.

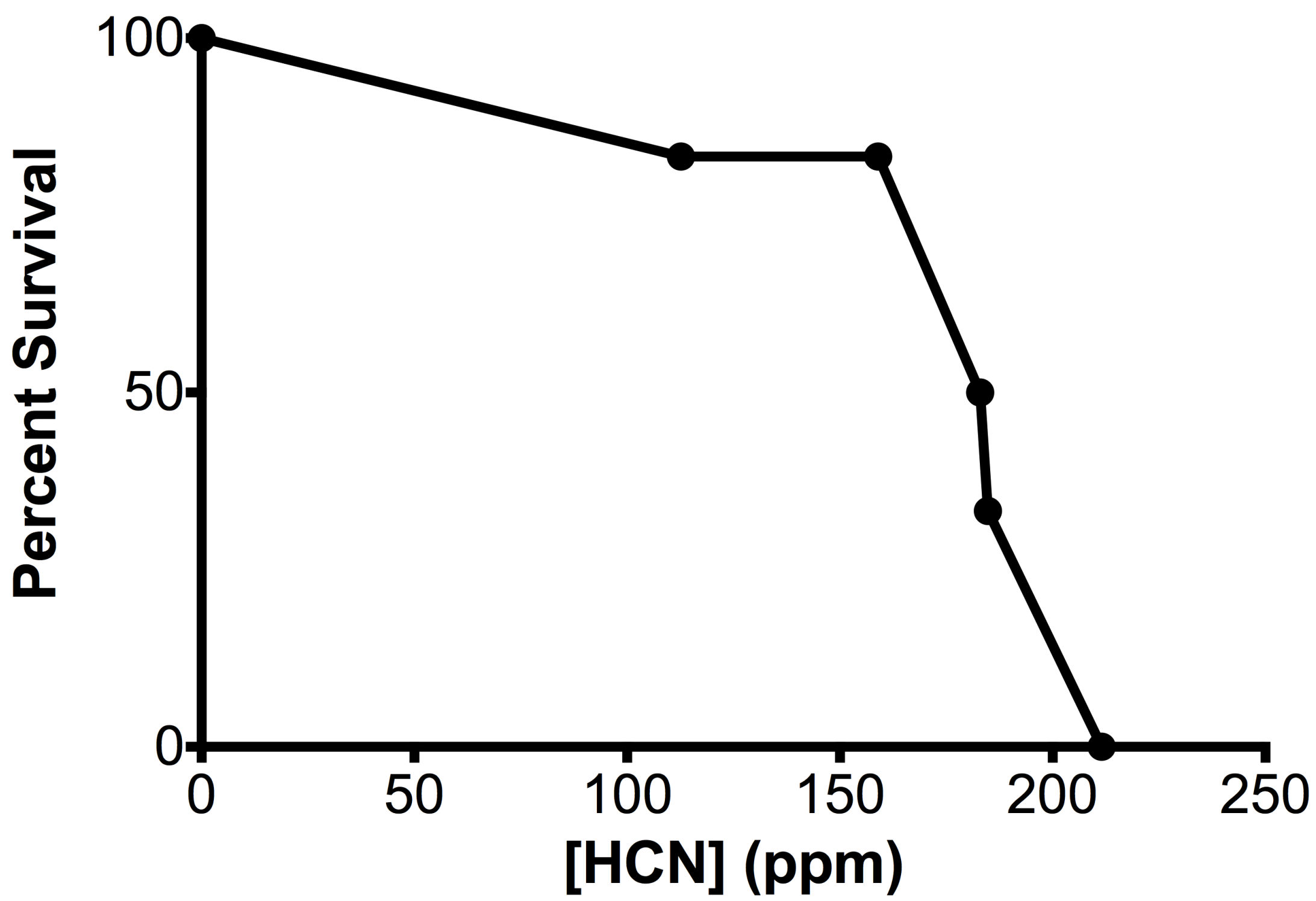

Supplement: Supplementary file 1 [file 775_2026_2153_MOESM1_ESM.pdf]
